# Transcranial pulse stimulation (TPS) improves depression in AD patients on state‐of‐the‐art treatment

**DOI:** 10.1002/trc2.12245

**Published:** 2022-02-10

**Authors:** Eva Matt, Gregor Dörl, Roland Beisteiner

**Affiliations:** ^1^ Department of Neurology Medical University of Vienna Vienna Austria

**Keywords:** Alzheimer's disease, brain stimulation, depression, functional connectivity, functional magnetic resonance imaging, transcranial pulse stimulation, ultrasound

## Abstract

**Introduction:**

Ultrasound‐based brain stimulation is a novel, non‐invasive therapeutic approach to precisely target regions of interest. Data from a first clinical trial of patients with Alzheimer's disease (AD) receiving 2‐4 weeks transcranial pulse stimulation (TPS) have shown memory and cognitive improvements for up to 3 months, despite ongoing state‐of‐the‐art treatment. Importantly, depressive symptoms also improved.

**Methods:**

We analyzed changes in Beck Depression Inventory (BDI‐II) and functional connectivity (FC) changes with functional magnetic resonance imaging in 18 AD patients.

**Results:**

We found significant improvement in BDI‐II after TPS therapy. FC analysis showed a normalization of the FC between the salience network (right anterior insula) and the ventromedial network (left frontal orbital cortex).

**Discussion:**

Stimulation of areas related to depression (including extended dorsolateral prefrontal cortex) appears to alleviate depressive symptoms and induces FC changes in AD patients. TPS may be a novel add‐on therapy for depression in AD and as a neuropsychiatric diagnosis.

## 1 INTRODUCTION

Recently, the novel therapeutic concept of ultrasound‐based brain stimulation has been introduced as a promising clinical add‐on therapy.^1^ With navigated ultrasound techniques (focused ultrasound [FUS], transcranial pulse stimulation [TPS]) neuromodulation is no longer limited to superficial brain areas but allows 3D targeting of deep areas of the human brain.^2^ The small ultrasound foci are independent from pathological conductivity changes and therefore brain areas can be targeted with unprecedented precision. First clinical studies with navigated ultrasound have shown cognitive improvements in Alzheimer's disease (AD) patients already receiving state‐of‐the‐art treatment.^3^, ^4^ In patients with chronic disorders of consciousness, improved clinical responsiveness has been reported.^5^, ^6^ In addition, reduction of cortical atrophy in AD core areas has been described after TPS treatment.^7^ While the exact cellular mechanisms of ultrasound‐induced neuromodulation are still under research, stimulation likely has an effect on cell membranes and mechanosensitive ion channels that further influence transmitter and neurotrophic factor concentrations and induce neuroplastic changes.^8^

In AD patients, depression is a major and particularly problematic comorbidity. Therapeutic effects of anti‐depressive medication are often limited and novel therapeutic approaches that may work as add‐on therapy are urgently needed. Here, we provide the very first data on such a possible new add‐on therapy. We investigate anti‐depressive effects of the novel clinically approved TPS therapy. In our previous multicenter AD study, improvements in depression scores were reported for up to 3 months after receiving stimulation.^3^ A specific subanalysis of neuropsychological and functional imaging data from this first clinical study with navigated ultrasound is presented.

## 2 METHODS

### 2.1 Patients

We included 20 patients from our previous study^3^ for whom functional magnetic resonance imaging (fMRI) data were available. Clinical AD was diagnosed according to International Classification of Diseases 10th revision (F00) and National Institute on Aging criteria. While most patients suffered from mild to moderate AD with a Mini‐Mental State Examination (MMSE) score of ≥18, an MMSE cutoff was not implemented to minimize inclusion criteria and heighten patient variability (mean MMSE = 20.94, standard deviation = 5.8, range = 6–30). Specifically, inclusion criteria were clinically stable patients with probable AD, at least 3 months of stable antidementia therapy (if any), age ≥18, signed informed consent. Exclusion criteria were noncompliance with the protocol, relevant intracerebral pathology unrelated to AD (e.g., brain tumor), hemophilia or blood clotting disorders or thrombosis, corticosteroid treatment within the last 6 weeks before first treatment. After dropout, 18 patients with fMRI completed the study (informed consent was obtained).

### 2.2 Study design

Patients received TPS treatment for 4 weeks, with three sessions per week (except for three patients for only 2 weeks, one patient for 3 weeks). MRI data acquisition and neuropsychological tests were performed the week before and after TPS therapy.

### 2.3 TPS treatment and regions of interest

TPS generates single ultrashort pulses with a broad frequency spectrum that can be administered with a repetition frequency range of 1 to 8 Hz^3^. Single ultrasound pressure pulses were applied using a NEUROLITH TPS generator (Storz Medical AG): duration about 3 μs, 0.2 mJ mm^−2^ energy flux density, pulse repetition frequency 5 Hz, 6000 pulses per session. Individual regions of interest (ROIs) were defined by a neurologist (R.B.) to target brain areas relevant to AD. These included the classical AD and depression stimulation target dorsolateral prefrontal cortex, areas of the memory (including default mode network) and language networks. Specifically, ROIs comprised: bilateral frontal cortex (dorsolateral prefrontal cortex and inferior frontal cortex extending to Broca's area, 2 × 800 pulses per hemisphere), bilateral lateral parietal cortex (extending to Wernicke's area, 2 × 400 pulses per hemisphere), and extended precuneus cortex (2 × 600 pulses). As previously described,^3^ individual real time tracking allowed standardized focal brain stimulation across the study participants.

### 2.4 MRI parameter

MRI sequences were acquired using a 3 T SIEMENS PRISMA MR with a 64‐channel head coil. For anatomical navigation scans, a T1‐weighted structural image was recorded using a MPRAGE sequence (TE/TR = 2.7/1800 ms, inversion time = 900 ms, flip angle = 9°, resolution 1 mm isotropic). For functional images, a T2*‐weighted EPI sequence was used, with 38 slices aligned to AC‐PC and covering the whole brain (TE/TR = 30/2500 ms, flip angle = 90°, in‐plane acceleration = GRAPPA 2, FOV = 230 × 230 mm, voxel size = 1.8 × 1.8 × 3 mm, 25% gap). Two hundred fifty volumes (10 minutes 25 seconds) for resting state fMRI were recorded.

**RESEARCH IN CONTEXT**

1. **Systematic review**: Depression is a widespread comorbidity of Alzheimer's disease (AD). Our previous and very first AD study indicated that ultrasound‐based brain stimulation further improves memory, cognitive functions, and network connectivity in patients already on state‐of‐the‐art treatment. Intriguingly, also depression scores improved up to 3 months. The present study performs a detailed subanalysis of possible antidepressive transcranial pulse stimulation (TPS) effects based on neuropsychological and functional imaging data. No similar study on antidepressive effects of ultrasound in AD is available (PubMed search).
2. **Interpretation**: Results indicate a specific antidepressive effect of TPS brain stimulation, which is based on a normalization of functional connectivity between key networks for depression.
3. **Future directions**: We provide first evidence that TPS brain stimulation is an effective tool as add‐on therapy for AD patients on state‐of‐the‐art treatment. Future studies with precisely targeted TPS may open novel avenues for add‐on effects on neuropsychiatric symptoms.

### 2.5 Behavioral assessments

For detailed analysis of depressive symptoms and correlation with fMRI results, the Beck Depression Inventory (BDI‐II) was used. BDI‐II values were not normally distributed according to Kolmogorov‐Smirnov test and were thus analyzed using the nonparametric Wilcoxon test for two paired variables (SPSS v24).

### 2.6 Functional connectivity analysis

For the resting state data analysis, all preprocessing procedures and analyses were performed using the CONN toolbox v19c.^9^ The CONN default preprocessing comprised realignment, unwarping, slice‐time correction, band segmentation, normalization, outlier detection, and smoothing (8 mm full width half‐maximum kernel). Subsequently, data were denoised using a band pass filter (0.008 to 0.09 Hz), removing motion confounds (six motion parameters and their first derivatives), applying aCompCor^10^ and scrubbing. Bivariate correlations of the corrected time series of all voxels were calculated for first‐level analysis. On group level, an ROI‐to‐ROI analysis was performed including all 164 default ROIs in the CONN toolbox (Harvard‐Oxford cortical and subcortical ROIs and network ROIs derived from the HCP dataset). Based on fMRI depression literature affected key networks are the default mode network, the ventromedial network, and the salience network.^11^ The post‐stimulation session was compared to the baseline using the connection threshold 0.05 p‐FDR corrected, combined with a multi‐voxel pattern analysis omnibus test on ROI level (cluster threshold 0.05 p‐uncorrected). Individual functional connectivity (FC) values of significant ROI‐to‐ROI connections (post‐stimulation vs. baseline) were correlated with BDI‐II scores using non‐parametric Spearman rank correlation analysis.

## 3 RESULTS

### 3.1 Depression scores

**FIGURE 1:**
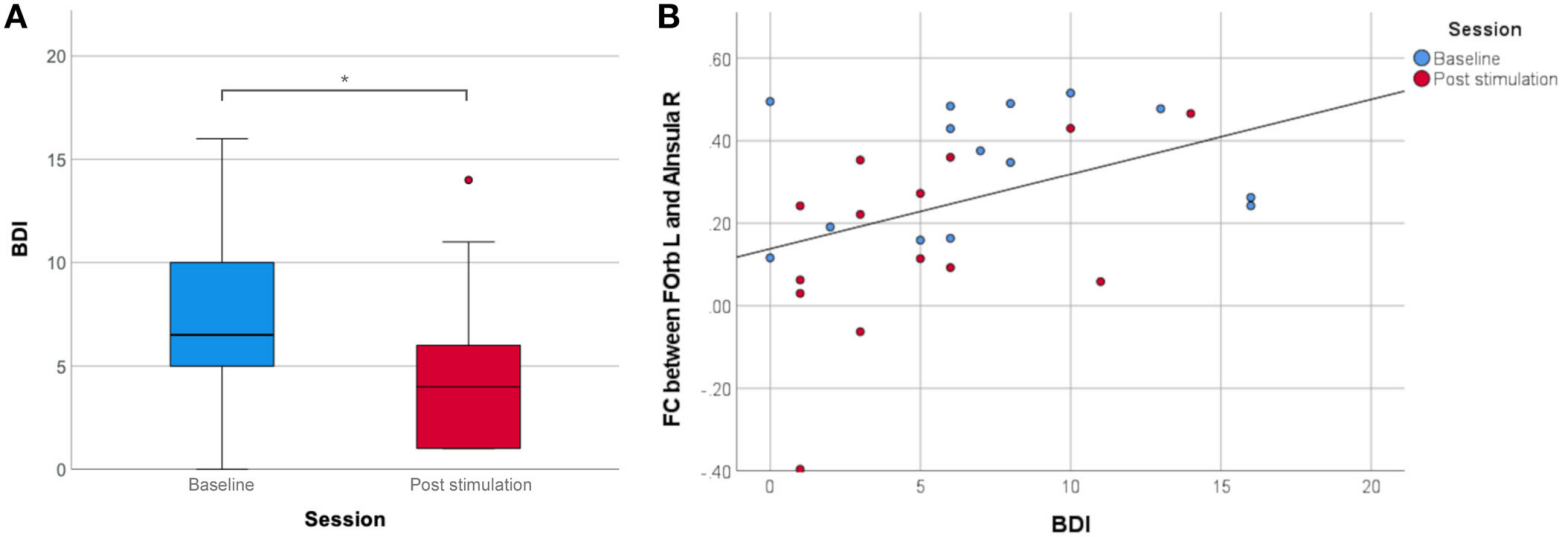
Depression reduction and functional connectivity correlation. A, Beck Depression Inventory (BDI) score before and after transcranial pulse stimulation (TPS). Depressive symptoms improved significantly (* *P* < .05) after the stimulation. B, Correlation between BDI‐II depression score and region of interest (ROI)‐to‐ROI functional connectivity (FC) between left frontal orbital cortex (FOrb L) and right anterior insula (AInsula R). Data for the baseline are depicted in blue and for the post‐stimulation sessions after the TPS interventions in red. The significant positive correlation (rho = .434, *P* = .021, N = 28) indicates that increased FC between these ROIs corresponds to more severe depressive symptoms

Out of 18 patients included in this study, 14 were able to complete the BDI‐II questionnaires in both sessions. On average, BDI‐II score was 7.36 (standard deviation [SD] = 5.09, N = 14) at baseline and 5.00 (SD = 4.11, N = 14) in the post‐stimulation session (Figure 1A). Comparison between both sessions using the non‐parametric Wilcoxon test revealed a significant improvement of depressive symptoms after TPS interventions (*P* = .037, two‐tailed).

### 3.2 Functional connectivity

**FIGURE 2:**
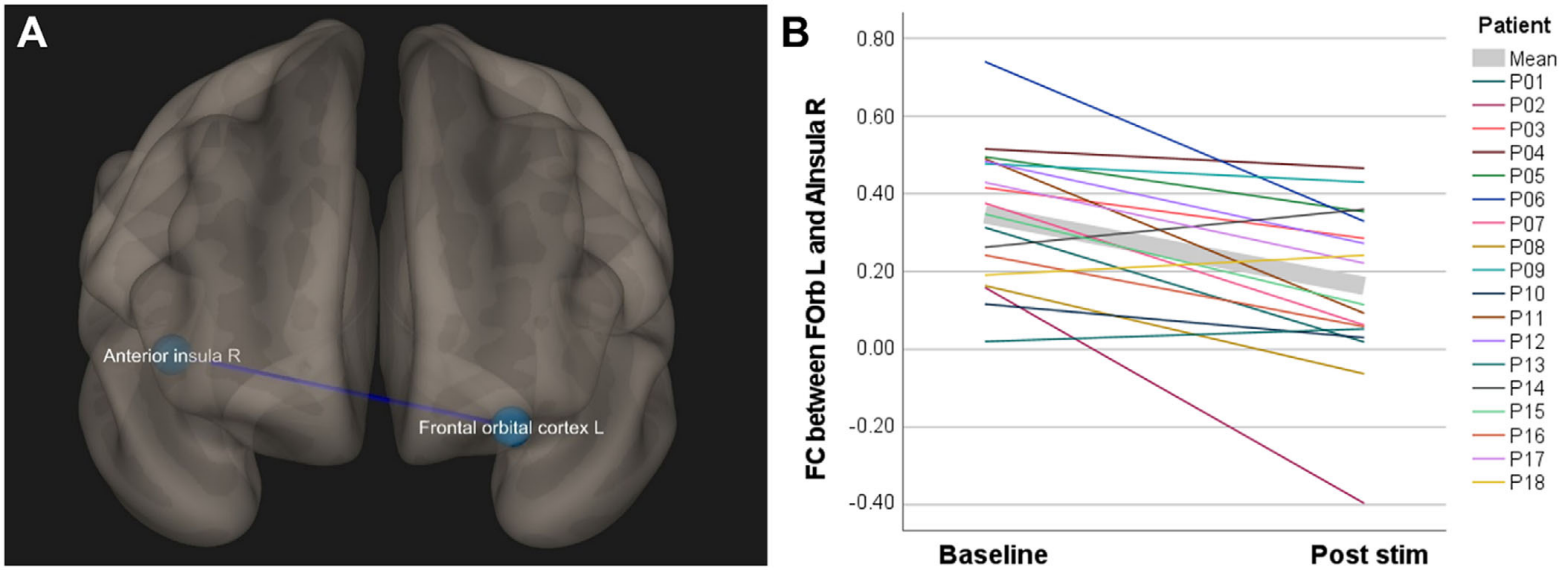
Functional connectivity changes after transcranial pulse stimulation (TPS) treatment in Alzheimer's disease patients. A, Region of interest (ROI) functional connectivity (FC). ROI‐to‐ROI analysis resulted in a significantly lower FC between the left frontal orbital cortex (FOrb L) and the right anterior insula (AInsula R) in the post stimulation session compared to the baseline. B, Individual FC between FOrb L and AInsula R in the baseline and in the post‐stimulation session with the mean values marked in gray. At baseline, the FC values of all patients were positive, but after the stimulation the FC values decreased in 15 out of 18 patients

ROI‐to‐ROI FC analysis using all default CONN ROIs revealed a single significant result: TPS treatment reduced FC between the left frontal orbital cortex (FOrb L; part of the ventromedial network) and the right anterior insula (AInsula R; part of the salience network defined by the CONN toolbox, Figure 2A). Intriguingly, all patients displayed a positive FC between these ROIs at baseline (Figure 2B). To elucidate FC characteristics between the FOrb L and the AInsula R in a healthy sample, the meta‐analysis tool Neurosynth (based on resting state FC data of 1000 healthy subjects) was used.^12^ In contrast to our patients, the resulting Neurosynth FC map of the FOrb L (Montreal Neurological Institute coordinates X = –44, Y = 34, Z = –12) showed a negative FC to the AInsula R as normal situation (peak FC: r = –0.24).

### 3.3 Correlation analysis

FC values between the left FOrb L and the right AInsula R were positively correlated with the BDI‐II score (rho = .434, *P* = .021, N = 28, Figure 1B). Higher FC values between these ROIs ( = higher disruption of normal connectivity) corresponded to more pronounced depressive symptoms.

## 4 DISCUSSION

We introduce a possible novel add‐on therapy for depression in AD patients based on navigated ultrasound pulses (TPS). Stimulation of multiple brain areas, including extended dorsolateral prefrontal cortex, led to a significant improvement in BDI‐II evaluations. Considering mood improvements in healthy subjects^13^ and depressed students,^14^ there now are several lines of evidence that precisely 3D‐navigated ultrasound may become a valuable add‐on therapy for depression. This will offer an additional chance for AD patients, already on state‐of‐the‐art treatment (i.e., anti‐dementia medication, cognitive training, occupational and physical therapy, dietary measures, etc.).

Corresponding to improvement of AD depression, FC data showed a significant connectivity normalization between the ventromedial network (VMN) and the salience network (SN). The VMN and SN have been proposed as relevant networks regarding depression and have been shown to form anti‐networks, that is, negatively correlated networks.^11^ This typical negative correlation in healthy persons has also been confirmed for FOrb L and AInsula R by using the FC meta‐analysis tool Neurosynth.^12^ A likely hypothesis therefore is that due to AD depression, the negative correlation between these areas was disrupted in our AD patients but improved after TPS. This was evident in the comprehensive ROI‐to‐ROI analysis as well as in our single patient data; in 15 out of 18 patients the correlation coefficient decreased, indicating a trend toward the typical negative FC. The remaining three patients, showing an increase in FC, can potentially be viewed as atypical responders. Future studies may clarify individual response patterns. Current understanding of functional networks in depression is still incomplete^15^ and is even less clear for depression as a comorbidity in AD. Our observation provides a possible functional basis for depression improvement in AD.

This is the first demonstration of ameliorating depressive symptoms in AD patients using ultrasound stimulation; however, there are limitations to be considered. There was no sham stimulation as a control condition. Nevertheless, the long‐term course of BDI improvements as well as the specificity of network changes render a pure placebo effect unlikely.^16^ Additionally, our previous neuropsychological and functional data showed that treatment response was confined to stimulated areas.^3^ Concerning treatment duration, our study stimulated over a course of 2 to 4 weeks. Future studies may want to investigate longer treatment periods as well as long‐term functional outcomes. Further, the small sample size (though comparable to other recent work in this field^14^, ^17^) limits any premature conclusions on the generalizability of the findings.

In conclusion, we present evidence that ultrasound stimulation may be a relevant add‐on treatment option for depressive symptoms in AD and possibly also in depression. For patients already on optimized pharmacological therapy, this holds the possibility to improve, besides memory functions, also depressive symptoms and increase quality of life. While promising, further investigations are needed to better understand stimulation effects on the functional basis of depressive symptoms.

## CONFLICTS OF INTEREST

This work was supported by research grants from STORZ Medical (including equipment, to R.B). R.B. is President of the Organization for Human Brain Mapping Alpine Chapter and the Austrian Society for fMRI (unpaid). E.M. received travel grants from the Austrian Research Association (ÖFG). G.D. has nothing to declare.

## References

[1] Beisteiner R , Lozano A . Transcranial ultrasound innovations ready for broad clinical application. Adv Sci. 2020;7(23):2002026. 10.1002/advs.202002026 PMC7709976 33304757

[2] Meng Y , Hynynen K , Lipsman N . Applications of focused ultrasound in the brain: from thermoablation to drug delivery. Nat Rev Neurol. 2021;17(1):7‐22. 33106619 10.1038/s41582-020-00418-z

[3] Beisteiner R , Matt E , Fan C , et al. Transcranial pulse stimulation with ultrasound in Alzheimer's disease—a new navigated focal brain therapy. Adv Sci. 2019;7:1902583. 10.1002/advs.201902583 PMC7001626 32042569

[4] Jeong H , Im J , Park J , et al. A pilot clinical study of low‐intensity transcranial focused ultrasound in Alzheimer's disease. Ultrasonography. 2021. [Epub]. 10.14366/usg.20138 PMC8446491 33730775

[5] Monti M , Schnakers C , Korb AS , Bystritsky A , Vespa PM . Non‐invasive ultrasonic thalamic stimulation in disorders of consciousness after severe brain injury: a first‐in‐man report. Brain Stimul. 2016;9(6):940‐941. 27567470 10.1016/j.brs.2016.07.008

[6] Cain JA , Spivak NM , Coetzee JP , et al. Ultrasonic thalamic stimulation in chronic disorders of consciousness. Brain Stimul. 2021;14(2):301‐303. 33465497 10.1016/j.brs.2021.01.008

[7] Popescu T , Pernet C , Beisteiner R . Transcranial ultrasound pulse stimulation reduces cortical atrophy in Alzheimer's patients: a follow‐up study. Alzheimer's Dement. 2021;7:e12121. 10.1002/trc2.12121 PMC7906128 33681449

[8] Tyler WJ , Lani SW , Hwang GM . Ultrasonic modulation of neural circuit activity. Curr Opin Neurobiol. 2018;50:222‐231. 29674264 10.1016/j.conb.2018.04.011

[9] Whitfield‐Gabrieli S , Nieto‐Castanon A . Conn: a functional connectivity toolbox for correlated and anticorrelated brain networks. Brain connect. 2012;2(3):125‐141. 22642651 10.1089/brain.2012.0073

[10] Behzadi Y , Restom K , Liau J , Liu TT . A component based noise correction method (CompCor) for BOLD and perfusion based fMRI. Neuroimage. 2007;37(1):90‐101. 17560126 10.1016/j.neuroimage.2007.04.042 PMC2214855

[11] Dunlop K , Hanlon CA , Downar J . Noninvasive brain stimulation treatments for addiction and major depression. Ann N Y Acad Sci. 2017;1394(1):31‐54. 26849183 10.1111/nyas.12985 PMC5434820

[12] Yarkoni T , Poldrack RA , Nichols TE , Van Essen DC , Wager TD . Large‐scale automated synthesis of human functional neuroimaging data. Nat Methods. 2011;8(8):665‐670. 21706013 10.1038/nmeth.1635 PMC3146590

[13] Sanguinetti JL , Hameroff S , Smith EE , et al. Transcranial focused ultrasound to the right prefrontal cortex improves mood and alters functional connectivity in humans. Front Hum Neurosci. 2020;14:52. 32184714 10.3389/fnhum.2020.00052 PMC7058635

[14] Reznik SJ , Sanguinetti JL , Tyler WJ , Daft C , Allen JJ . A double‐blind pilot study of transcranial ultrasound (TUS) as a five‐day intervention: tUS mitigates worry among depressed participants. Neurol Psychiatry Brain Res. 2020;37:60‐66.

[15] Brakowski J , Spinelli S , Dörig N , et al. Resting state brain network function in major depression. Depression symptomatology, antidepressant treatment effects, future research. J Psychiatr Res. 2017;92:147‐159. 28458140 10.1016/j.jpsychires.2017.04.007

[16] Ito K , Corrigan B , Romero K , et al. Understanding placebo responses in Alzheimer's disease clinical trials from the literature meta‐data and CAMD database. J Alzheimer's Dis. 2013;37(1):173‐183. 23803296 10.3233/JAD-130575

[17] Cui H , Ren R , Lin G , et al. Repetitive transcranial magnetic stimulation induced hypoconnectivity within the default mode network yields cognitive improvements in amnestic mild cognitive impairment: a randomized controlled study. J Alzheimer's Dis. 2019;69(4):1137‐1151. 31127779 10.3233/JAD-181296

